# Uterine Dilator

**Published:** 1877-07-20

**Authors:** 


UTERINE DILATOR.

Dr. Serley, of Illinois, exhibited a new uterine dilator. It consisted of a silk bag outside of a rubber sack. A tube is carried into the sack to its fundus for the purpose of holding a metallic sound, by means of which the dilator is held within the cervical cavity to be dilated. A second tube connects the dilator with the syringe, by means of which the instrument does not slip out of the canal, as Barnes' dilator is inclined to do.

